# The Effect of Tourniquet Deflation Timing on Outcomes of Common Upper Extremity Surgeries

**DOI:** 10.7759/cureus.99373

**Published:** 2025-12-16

**Authors:** Matthew A Peterman, Andrea H Johnson, Faith I Wheeler, Caroline Donegan, Sydney Holmes, Jane C Brennan, Justin Turcotte, Risa Reid, Christopher Jones, Jeffrey Gelfand, Alexander Shushan

**Affiliations:** 1 Orthopedic Research, Anne Arundel Medical Center, Annapolis, USA; 2 Orthopedics, Anne Arundel Medical Center, Annapolis, USA; 3 Orthopedic and Surgical Research, Anne Arundel Medical Center, Annapolis, USA; 4 Orthopedic Surgery, Anne Arundel Medical Center, Annapolis, USA

**Keywords:** distal radius fracture orif, endoscopic carpal tunnel release, open carpal tunnel release, patient-reported outcome measures, postoperative complications, postoperative opioid use, postoperative pain, tourniquet use, trigger finger release

## Abstract

Background

Historically, upper extremity procedures have relied on tourniquets to achieve hemostasis and provide a bloodless surgical field. However, a shift away from routine tourniquet use occurred in the early 2000s with the popularization of the wide-awake local anesthesia with no tourniquet (WALANT) technique. Despite this trend, many surgeons still prefer pneumatic tourniquets, which may improve visualization and operative efficiency. The purpose of this study was to compare early postoperative pain and function between procedures where the tourniquet was deflated after final wound closure and before final closure in carpal tunnel release (CTR), trigger finger (TF) release, and open reduction and internal fixation (ORIF) for distal radius fracture (DRF) cohorts.

Methods

A retrospective study of 150 patients undergoing CTR, 150 patients undergoing TF release, and 150 patients undergoing ORIF of a DRF from 2023 to 2025 was performed. Within each procedure category, patients were grouped by whether the tourniquet was deflated before or after final closure. Differences in demographics, comorbidities, perioperative details, and 30-day outcomes were assessed using univariate analysis. Multivariate linear and logistic regression were used to assess tourniquet deflation after closure as a predictor of outcomes. Statistical significance was assessed at p<0.05.

Results

Overall, demographics and comorbidities were largely similar between groups for the procedures evaluated. In the post-anesthesia care unit (PACU), the deflation after closure group had a longer time in recovery for both CTR and TF (both p < 0.001). CTR patients in the deflation after closure group reported higher average pain scores in the PACU (0.8 ± 2.0 vs. 0.3 ± 1.1; p = 0.026) and were more likely to receive opioids in the PACU (14.4 vs. 1.7%; p = 0.009). These differences in pain during early recovery were not observed in the TF or DRF cohorts. In CTR, after controlling for body mass index, operating room (OR) location, and endoscopic release, the deflation after closure group was significantly more likely to receive opioids in recovery (OR: 9.03; p = 0.043), but no significant differences in other PACU outcomes were observed. In TF, after controlling for race and OR location, the deflation after closure group had increased time in recovery (β: 10.44 min; p < 0.001) and decreased maximum pain score in the PACU (β: -1.11; p = 0.026). Additionally, the TF deflation after closure group was less likely to report a pain score of 5+ in recovery (OR: 0.32; p = 0.028). No significant differences in rates of nerve injury, surgical site infection (SSI), wound issues, pain complaints, or QuickDASH scores at 30-day follow-up were observed between groups for any of the three procedures.

Conclusion

This study's findings demonstrate that the timing of tourniquet deflation, either before closure or after closure, does not significantly impact the majority of early postoperative clinical outcomes or patient-reported outcome measures following CTR, TF, and DRF. These findings suggest that both deflation timings are safe for patients undergoing these procedures. The choice of when to release the tourniquet can be guided by surgeon preference and the specific context of the procedure. Further study is warranted to identify optimal tourniquet deflation timing protocols in common upper extremity surgeries.

## Introduction

Carpal tunnel release (CTR), trigger finger (TF) release, and distal radius fracture (DRF) fixation are three of the most frequently performed procedures in upper extremity surgery [1-3]. Carpal tunnel syndrome (CTS) is a common acquired compressive neuropathy of the median nerve within the carpal tunnel reducing intraneural blood supply, which presents with numbness and tingling in the median nerve distribution of the hand [4,5]. CTS is very common, estimated to be present in between 1% and 3% of the population in the United States [6,7]. TF is a multifactorial disease characterized by the painful popping or clicking of a tendon in the digit as it slides through pulleys, most commonly the A1 pulley, during flexion and extension of the affected finger [8,9]. TF is also very common, estimated to affect between 2% and 3% of the general population in the United States [8]. Finally, DRFs are one of the most common of all orthopedic injuries with over 100,000 DRF insurance claims annually from 2005 to 2014 in the United States. DRFs account for almost 20% of all fractures seen by physicians and are particularly prevalent in the elderly population [10,11]. Broadly, all three of these conditions frequently have favorable outcomes when managed surgically [8,12-14]. Traditionally, all three surgeries have been conducted utilizing pneumatic arm tourniquets to control hemostasis during surgery, given the rich vasculature of the hand and the need for visualization during surgery [1,3,7,15]. As these procedures have evolved, alternative approaches to hemostasis have been developed in efforts to improve patient outcomes and reduce pain experienced during and after the said procedures [1,2,14].

Historically, upper extremity procedures have relied on tourniquets of different types to achieve hemostasis and provide a bloodless surgical field [15,16]. This practice was expanded to use during elective operative procedures by Joseph Lister in 1864 and modernized with Harvey Cushing's pneumatic device in 1904 [15,16]. Cushing developed a pneumatic tourniquet utilizing compressed air for hemostasis, becoming the basis for the modern pneumatic tourniquet used globally today [16]. The tourniquet became an essential tool in hand surgery due to the hands' rich vascularity which causes challenges in achieving adequate visualization during surgery without occluding blood flow [15]. However, a shift away from routine tourniquet use occurred in the early 2000s with the popularization of the wide-awake local anesthesia with no tourniquet (WALANT) technique, popularized by Dr. Donald Lalonde [2,15,17]. By eliminating the tourniquet, WALANT removes the primary source for conscious intraoperative pain, rendering sedation no longer required during those surgeries [2,3,13]. These changes avoid potential complications associated with tourniquet use as well as sedation, such as postoperative pain and delayed recovery [2,3,13]. Randomized controlled trials (RCTs) have demonstrated equivalent long-term outcomes for patients undergoing WALANT when compared with traditional hemostasis protocols, with no increase in long-term complications observed, leading to widespread adoption globally [2,15,16].

Despite the advantages of WALANT, many surgeons still prefer pneumatic tourniquets to ensure a completely bloodless field, which may improve visualization and operative efficiency by reducing time spent managing hemostasis with electrocautery [13,15,18]. Other purported benefits include potentially reducing the likelihood of iatrogenic injury to nerves or other present structures during dissection due to improved visualization [19,20]. These benefits must however be weighed against potential risks of tourniquet use, such as intraoperative tourniquet site pain and post-deflation tourniquet site discomfort [19]. While the choice between using a tourniquet and the WALANT technique in upper extremity surgery has been studied extensively, a more nuanced aspect of tourniquet management in the precise timing of deflation remains under-investigated [19,21]. Factors contributing to the severity and nature of tourniquet-site pain have been explored, including tourniquet duration, inflation pressure, cuff width and design, and tourniquet location [19,21]. Overall inflation duration has emerged as the most consistently reported factor, with duration directly correlating with perceived pain in multiple studies [18,19]. To date, few studies have specifically evaluated the timing of tourniquet deflation, i.e., before full closure or after closure [7]. The purpose of this study was to compare early postoperative pain and function between procedures where the tourniquet was deflated after final wound closure and before final closure in CTR, TF release, and open reduction and internal fixation (ORIF) for DRF cohorts. We hypothesized that earlier tourniquet deflation may reduce early postoperative pain and risk for tourniquet-related complications.

## Materials and methods

This study was deemed exempt by the WCG Institutional Review Board (IRB).

Study population

A retrospective study of 150 patients undergoing CTR, 150 patients undergoing TF release, and 150 patients undergoing ORIF of a DRF from July 2023 to April 2025 was performed. Surgeries were performed by four upper extremity fellowship-trained surgeons at two acute care hospitals, namely, Luminis Health Anne Arundel Medical Center and Luminis Health Doctors Community Medical Center, and one hospital-affiliated ambulatory surgery center (ASC), Luminis Health Anne Arundel Medical Center Outpatient Surgery Center. All patients who underwent one of the above procedures and had at least one postoperative follow-up visit were included in the study. Patients were grouped by whether the tourniquet was deflated before or after wound closure.

Independent variables

Demographics and comorbidities of interest were age, sex, body mass index (BMI), race, and the Charlson Comorbidity Index (CCI) score. Perioperative details assessed were minutes in the operating room (OR), tourniquet time, and OR location (hospital vs. ASC). For CTR, endoscopic vs. open release was also assessed.

Outcomes

Immediate postoperative outcomes evaluated in the post-anesthesia care unit (PACU) included minutes in recovery, maximum pain score, rates of pain scores ≥5, and whether patients received opioids. Pain scores were reported on the numeric rating scale (NRS). Thirty-day outcomes of interest included nerve injury, surgical site infection (SSI), wound issues (i.e., maceration, bleeding, dehiscence, drainage), and pain complaints. Follow-up was assessed in days, and minimum two-week and 30-day follow-up rates were evaluated. Patient-reported outcome measures (PROM) using the Quick Disabilities of the Arm, Shoulder, and Hand (QuickDASH) tool were assessed preoperatively and 30-day postoperatively [22]. Improvement in QuickDASH scores was measured as the change in preoperative to postoperative scores.

Power analysis

A priori power analysis was conducted to determine whether this study had adequate sample sizes to detect medium and large effect sizes for continuous and categorical endpoints, respectively, with an 80% power. Cohen's d was defined as the difference between two means divided by the standard deviation of the data. Cohen described a "d" of 0.20 to be a small effect size, 0.50 to be a medium effect size, and 0.80 to be a large effect size for continuous endpoints. Cohen's w was defined as a measure of effect size used for chi-squared tests. Cohen described a "w" of 0.10 to be a small effect size, 0.30 to be a medium effect size, and 0.50 to be a large effect size for categorical endpoints [23]. The total sample sizes necessary to detect large effect sizes were 52 and 64 for continuous and categorical endpoints, respectively. A total sample size of 128 and 176 patients was required to detect a medium effect size. Therefore, the study sample size of 150 patients per procedure type was deemed sufficient to detect medium or large effects in continuous outcome measures and large effects in categorical measures.

Statistical analysis

Differences in demographics, comorbidities, perioperative details, and postoperative and patient-reported outcomes were assessed using univariate analysis (two-sided independent samples t-tests and chi-squared tests). Fisher's exact test was used when the assumptions of the chi-squared test were not met. Multivariate linear and logistic regression were used to assess tourniquet deflation after wound closure as a predictor of recovery metrics in CTR and TF controlling for significant differences between groups. Multivariate analysis of DRF outcomes was not performed, as no statistically significant differences in outcomes were observed in the univariate analysis. All statistical analyses were performed using R Studio (Version 4.2.2; 2009-2025; Posit Software, Boston, MA, USA). Statistical significance was assessed at p < 0.05. 

Study funding

This study received no funding.

## Results

Overall, demographics and comorbidities were largely similar between groups for the procedures evaluated, although some significant differences were noted. In CTR, patients whose tourniquet was deflated after closure had a higher BMI than those whose tourniquet was deflated before closure (32.6 ± 7.5 vs. 30.2 ± 5.8; p = 0.031). In TF, there was a significant difference in the distribution of race (p = 0.019), with minority races accounting for a greater proportion of patients in the deflation after closure group. In DRF, the after closure group on average was older (60.9 ± 16.9 vs. 52.5 ± 18.8; p = 0.005), had a higher proportion of White patients (81.2% vs. 59.3%; p = 0.003), and had an increased comorbidity burden as shown by the Charlson Comorbidity Index (CCI) score (2.3 ±1.9 vs. 1.6 ± 1.7; p = 0.023) (Table 1).

**Table 1:** Patient demographics and comorbidities Data is expressed as mean ± SD or n (%). P-values of <0.05 are highlighted in bold. * indicates Fisher's exact test. t indicates a two-sample t-test. χ^2^ indicates a chi-squared test for independence. Before/after closure denotes the timing of tourniquet deflation relative to wound closure. BMI: body mass index; CCI: Charlson Comorbidity Index

	Carpal tunnel release (n = 150)				Trigger finger (n = 150)				Distal radius fracture (n = 150)			
	Before closure (n = 60)	After closure (n = 90)	Test statistic	P-value	Before closure (n = 67)	After closure (n = 83)	Test statistic	P-value	Before closure (n = 81)	After closure (n = 69)	Test statistic	P-value
Age, years	61.8 ± 14.4	64.4 ± 13.7	t = -1.16	0.267	61.7 ± 9.3	63.8 ± 13.3	t = -1.16	0.246	52.5 ± 18.8	60.9 ± 16.9	t = -2.88	0.005
Sex												
Male	22 (36.7)	31 (34.4)	χ^2^= 0.01	0.917	19 (28.4)	27 (32.5)	χ^2^= 0.14	0.709	22 (27.2)	12 (17.4)	χ^2^= 1.51	0.219
Female	38 (63.3)	59 (65.6)			48 (71.6)	56 (67.5)			59 (72.8)	57 (82.6)		
BMI, kg/m^2^	30.2 ± 5.8	32.6 ± 7.5	t = -2.17	0.031	31.4 ± 7.1	30.2 ± 5.1	t = 1.18	0.239	28.0 ± 6.9	26.7 ± 5.3	t = 1.32	0.191
Race												
White	48 (80.0)	69 (76.7)	N/A	0.809*	51 (76.1)	50 (60.2)	N/A	0.019*	48 (59.3)	56 (81.2)	N/A	0.003*
Black	9 (15.0)	17 (18.9)			15 (22.4)	23 (27.7)			16 (19.8)	10 (14.5)		
Other	3 (5.0)	4 (4.4)			1 (1.5)	10 (12.0)			17 (21.0)	3 (4.3)		
CCI score	2.7 ± 2.2	2.8 ± 1.9	t = 0.24	0.811	2.3 ± 1.5	2.6 ± 1.8	t = -1.30	0.197	1.6 ± 1.7	2.3 ±1.9	t = -2.31	0.023

When evaluating OR metrics, the after closure group had increased operative time and tourniquet time for both CTR and TF (all p < 0.001). Additionally, the after closure group had fewer surgeries performed in the ASC in both CTR (54.4% vs. 88.3%; p < 0.001) and TF (86.7% vs. 97%; p = 0.038). In DRF patients, shorter operative times were observed when the tourniquet was deflated after wound closure (80.2 ± 20.7 vs. 99.7 ± 43.9 min; p < 0.001). In the PACU, the deflation after closure group had a longer time in recovery for both CTR and TF (both p < 0.001). CTR patients in the deflation after closure group reported higher average pain scores in the PACU (0.8 ± 2.0 vs. 0.3 ± 1.1; p = 0.026) and were more likely to receive opioids in the PACU (14.4 vs. 1.7%; p = 0.009). These differences in pain during early recovery were not observed in the TF or DRF cohorts (Table 2).

**Table 2:** Perioperative details Data is expressed as mean ± SD or n (%). P-values of <0.05 are highlighted in bold. * indicates Fisher's exact test. t indicates a two-sample t-test. χ^2^ indicates a chi-squared test for independence. Before/after closure denotes the timing of tourniquet deflation relative to wound closure. OR: operating room; ASC: ambulatory surgery center

	Carpal tunnel release (n = 150)				Trigger finger (n = 150)				Distal radius fracture (n = 150)			
Perioperative details	Before closure (n = 60)	After closure (n = 90)	Test statistic	P-value	Before closure (n = 67)	After closure (n = 83)	Test statistic	P-value	Before closure (n = 81)	After closure (n = 69)	Test statistic	P-value
Minutes in OR	27.0 ± 6.4	42.9 ± 24.2	t = -5.94	<0.001	23.5 ± 4.3	32.6 ± 11.4	t = -6.70	<0.001	99.7 ± 43.9	80.2 ± 20.7	t = 3.56	<0.001
Endoscopic procedure	59 (98.3)	82 (91.1)	N/A	0.086*	N/A	N/A		N/A	N/A	N/A		N/A
Tourniquet time, minutes	3.9 ± 3.1	19.0 ± 16.5	t = -8.43	<0.001	4.6 ± 1.7	15.0 ± 8.5	t = -10.98	<0.001	50.3 ± 27.7	52.1 ± 17.1	t = -0.49	0.628
OR location												
ASC	53 (88.3)	49 (54.4)	χ^2 ^= 17.48	<0.001	65 (97)	72 (86.7)	N/A	0.038*	56 (69.1)	50 (72.5)	χ^2 ^= 0.07	0.790
Hospital	7 (11.7)	41 (45.6)			2 (3.0)	11 (13.3)			25 (30.9)	19 (27.5)		
Minutes in recovery	39.2 ± 12.3	48.6 ± 17.7	t = -3.76	<0.001	22.5 ± 16.0	35.2 ± 14.8	t = -4.93	<0.001	54.7 ± 20.0	60.3 ± 38.8	t = -1.08	0.282
Max pain score	0.3 ± 1.1	0.8 ± 2.0	t = -2.25	0.026	1.9 ± 3.5	0.9 ± 2.3	t = 1.98	0.050	1.5 ± 2.9	1.1 ± 2.3	t = 1.08	0.279
Pain score 5+	2 (3.3)	9 (10.0)	N/A	0.201*	14 (20.9)	8 (9.6)	χ^2 ^= 2.91	0.088	15 (18.5)	7 (10.1)	χ^2^ = 1.47	0.225
Received opioids	1 (1.7)	13 (14.4)	N/A	0.009*	0 (0)	2 (2.4)	N/A	0.502*	15 (18.5)	11 (15.9)	χ^2 ^= 0.04	0.842

In CTR, after controlling for BMI, OR location, and endoscopic release, the after closure group was still significantly more likely to receive opioids in recovery (OR: 9.03; 95% CI: 1.55 to 172.20; p = 0.043), but no significant differences in other PACU outcomes were observed (Table 3).

**Table 3:** Multivariate linear and logistic regression: tourniquet removal as a predictor of recovery metrics in carpal tunnel release Controlling for body mass index, operating room location, and endoscopic carpal tunnel release. A p-value of <0.05 is highlighted in bold. After closure denotes the timing of tourniquet deflation relative to wound closure. OR: odds ratio indicating logistic regression; β: beta indicating linear regression

In recovery	After closure (β/OR)	95% CI	P-value
Minutes in recovery (β)	4.70	-0.49 to 9.88	0.075
Max pain score (β)	0.39	-0.21 to 0.98	0.199
Pain score 5+ (OR)	2.86	0.61 to 20.56	0.219
Received opioids (OR)	9.03	1.55 to 172.20	0.043

In TF, after controlling for race and OR location, the after closure group had increased time in recovery (β: 10.44 min; 95% CI: 5.44 to 15.44; p < 0.001) and maximum pain score in the PACU (β: -1.11; 95% CI: -2.09 to -0.13; p = 0.026). Additionally, the after closure group was less likely to receive opioids in recovery (OR: 0.32; 95% CI: 0.11 to 0.86; p = 0.028) (Table 4).

**Table 4:** Multivariate linear and logistic regression: tourniquet removal as a predictor of recovery metrics in trigger finger release Controlling for race and OR location. P-values of <0.05 are highlighted in bold. After closure denotes the timing of tourniquet deflation relative to wound closure. OR: odds ratio indicating logistic regression; β: beta indicating linear regression

In recovery	After closure (β/OR)	95% CI	P-value
Minutes in recovery (β)	10.44	5.44 to 15.44	<0.001
Max pain score (β)	-1.11	-2.09 to -0.13	0.026
Pain score 5+ (OR)	0.32	0.11 to 0.86	0.028

When evaluating 30-day outcomes, no significant differences in rates of nerve injury, SSI, wound issues, or pain complaints were observed between groups for any of the three procedures. QuickDASH scores were also similar between groups at baseline and 30-day follow-up for both groups in all procedures evaluated. The only exception to this was a greater severity of arm/shoulder/hand pain reported among DRF patients in the after closure group (3.1 ± 1.0 vs. 2.5 ± 0.8 (five-point scale); p = 0.019) at 30-day follow-up (Table 5).

**Table 5:** Thirty-day postoperative and patient-reported outcomes Data is expressed as mean ± SD or n (%). P-values of <0.05 are highlighted in bold. * indicates Fisher's exact test. t indicates a two-sample t-test. χ^2^ indicates a chi-squared test for independence. Before/after closure denotes the timing of tourniquet deflation relative to wound closure. SSI: surgical site infection; N/A: not applicable; QuickDASH: Quick Disabilities of the Arm, Shoulder, and Hand

	Carpal tunnel release (n = 150)				Trigger finger (n = 150)				Distal radius fracture (n = 150)			
	Before closure (n = 60)	After closure (n = 90)	Test statistic	P-value	Before closure (n = 67)	After closure (n = 83)	Test statistic	P-value	Before closure (n = 81)	After closure (n = 69)	Test statistic	P-value
Nerve injury	0 (0)	0 (0)	N/A	N/A	0 (0)	0 (0)	N/A	N/A	0 (0)	0 (0)	N/A	N/A
SSI	1 (1.7)	2 (2.2)	N/A	1*	0 (0)	1 (1.2)	N/A	1*	0 (0)	0 (0)	N/A	N/A
Skin issue	0 (0)	1 (1.1)	N/A	1*	1 (1.5)	2 (2.4)	N/A	1*	0 (0)	0 (0)	N/A	N/A
Pain complaint	4 (6.7)	7 (7.8)	N/A	1*	4 (6.0)	4 (4.8)	N/A	1*	1 (1.2)	1 (1.4)	N/A	1*
Days to follow-up	20.8 ± 46.7	24.1 ± 41.5	t = -0.44	0.659	24.5 ± 55.7	28.9 ± 31.1	t = -0.57	0.566	110.8 ± 77.4	117.4 ± 84.7	t = -0.50	0.620
Minimum 2-week follow-up	40 (66.7)	62 (68.9)	χ^2 ^= 0.01	0.915	40 (59.7)	63 (75.9)	χ^2 ^= 3.80	0.051	79 (97.5)	65 (94.2)	N/A	0.414*
Minimum 30-day follow-up	4 (6.7)	14 (15.6)	N/A	0.127*	6 (9.0)	23 (27.7)	χ^2 ^= 7.20	0.007	76 (93.8)	63 (91.3)	N/A	0.755*
Had QuickDASH at baseline	57 (95.0)	81 (90.0)	N/A	0.363*	49 (73.1)	55 (66.3)	χ^2 ^= 0.53	0.466	53 (65.4)	53 (76.8)	χ^2 ^= 1.81	0.178
Baseline QuickDASH score	42.8 ± 22.9	38.7 ± 22.8	t = 1.05	0.298	34.0 ± 19.1	30.6 ± 18.9	t = 0.90	0.369	81.3 ± 16.3	73.5 ± 24.7	t = 1.91	0.059
Severity of arm/shoulder/hand pain at baseline	2.9 ± 1.0	2.8 ± 1.1	t = 0.67	0.503	3.0 ± 0.9	3.0 ± 0.9	t = -0.44	0.661	3.9 ± 1.1	3.6 ± 1.2	t = 1.13	0.260
Had QuickDASH at 30-day post-op	36 (60.0)	35 (38.9)	χ^2 ^= 5.62	0.018	24 (35.8)	20 (24.1)	χ^2 ^= 1.93	0.165	39 (48.1)	22 (31.9)	χ^2 ^= 3.44	0.064
30-day post-op QuickDASH score	41.7 ± 21.2	35.7 ± 23.6	t = 1.13	0.264	32.6 ± 16.8	25.7 ± 16.0	t = 1.39	0.171	58.2 ± 21.9	68.9 ± 19.3	t = -1.97	0.054
Severity of arm/shoulder/hand pain at 30-day post-op	2.5 ± 0.8	2.4 ± 1.0	t = 0.71	0.480	2.5 ± 0.9	2.2 ± 0.8	t = 1.20	0.235	2.5 ± 0.8	3.1 ± 1.0	t = -2.45	0.019
Improvement in QuickDASH	0.82 ± 21.3	-0.58 ± 18.2	t = 0.29	0.770	5.5 ± 16.1	6.7 ± 15.3	t = -0.25	0.807	20.1 ± 27.3	7.6 ± 16.3	t = 1.87	0.069

## Discussion

The results of the current study suggest that while the timing of tourniquet deflation may affect perioperative throughput and early pain in the PACU, it does not appear to influence 30-day complication rates or PROMs after common upper extremity procedures. Specifically, our risk-adjusted analyses indicated that tourniquet deflation after closure is associated with higher rates of PACU opioid use in CTR patients and longer PACU recovery times but lower PACU pain scores in TF release patients. However, given the low pain scores and rates of opioid use observed across groups, the clinical significance of these differences is likely minimal. Based on these results, tourniquet deflation before or after wound closure appears to be a safe and viable alternative in patients undergoing CTR, TF release, or DRF repair.

This study's finding that tourniquet deflation timing does not significantly affect 30-day postoperative clinical outcomes or PROMs following CTR is largely consistent with existing literature [7]. A prospective randomized trial of 48 patients by Pai et al. compared releasing the tourniquet before versus after wound closure following CTR and found no statistically significant differences in key postoperative outcomes [7]. The authors likewise observed longer average tourniquet inflation time in their after closure cohort than their before closure cohort (10.6 vs. 6.3 minutes) [7]. In contrast to Pai et al.'s analysis, we observed longer recovery times, higher pain scores, and increased rates of opioid administration in the PACU among patients with the tourniquet deflated after closure group [7]. However, after controlling for BMI, OR location, and endoscopic surgery, only an association between deflation after closure and PACU opioid use remained, suggesting that other patient and systemic factors may have influenced the differences observed. Therefore, while both approaches to tourniquet deflation appear viable, surgeons may consider using more aggressive early multimodal analgesia to avoid reliance on opioids in the PACU, particularly in cases where the tourniquet is left inflated during wound closure. There is conflicting evidence on the impact of tourniquet use on patient discomfort during and following CTR in the current literature [1,24]. In a prospective randomized controlled trial of 50 patients by Ralte et al., the tourniquet group reported significantly more discomfort intraoperatively than the no-tourniquet group [1]. In contrast, a randomized controlled trial by Urias et al. found comparable symptom severity and functional status immediately postoperatively between CTRs performed with and without tourniquet as measured by a Levine-Katz Questionnaire scale [13]. In the broader context, the current literature shows a consensus with findings similar to long-term outcomes between CTRs performed with and without a tourniquet [13,15,25]. A randomized controlled trial by de Roo et al., on 142 patients, found no differences in 12-month QuickDASH and Boston Carpal Tunnel Questionnaire PROMs between patients following CTR performed with and without a tourniquet [15]. While our study only evaluated PROMs at 30 days postoperatively, our findings further support the conclusion that tourniquet use does not affect postoperative functional outcomes after CTR.

In line with studies on CTR, the literature concerning the effect of tourniquet use on patient outcomes after TF release procedures is mixed. A randomized controlled trial by Volkmar et al., on 66 CTR, ganglion excision, and TF procedures, divided patients into groups with an upper arm tourniquet, forearm tourniquet, and forearm tourniquet with immediate deflation following the procedure before closure [26]. The authors observed no significant differences in intraoperative pain, postoperative pain, or patient satisfaction among the three groups [26]. Contrasting with these findings, we observed significantly reduced pain scores and fewer patients reporting pain scores of 5 or higher in the PACU, but longer PACU stays in our deflation after closure cohort in the multivariate analysis. A retrospective cohort analysis of 47 patients by Gurich et al. on a variety of upper extremity procedures performed including TF evaluated the tourniquet inflation duration for each procedure. The average tourniquet time across all procedures, including CTR, TF, and ganglion excision, was 16 minutes, and the authors noted no significant complications or tourniquet-related complications in any patients, further supporting the safety of shorter tourniquet durations during TF release [27]. In line with these other studies, we observed no significant differences in complication rates between the different tourniquet protocols within our TF cohort.

More so than for CTR and TF, the use of a pneumatic tourniquet in surgical fixation for DRF is considered standard to create a bloodless field, enhance visualization, and limit blood loss [3,28]. A retrospective analysis by Ruckle et al. of 402 patients who underwent volar plating for DRF investigated the specific impact of tourniquet duration on immediate postoperative outcomes and found no correlation between tourniquet time and immediate postoperative opioid consumption as measured by morphine equivalent dose [24]. Stratifying tourniquet times also revealed no correlation with postoperative pain scores [24]. In congruence with this data, our study found no significant differences between groups following DRF in time spent in recovery, maximum pain score, or the number of patients who received opioids. In a review article by Oragui et al. on general tourniquet use in upper extremity surgery, the authors note that no "safe" tourniquet duration cutoff has been established to eliminate potential tourniquet-related complications such as ischemia and muscle dysfunction [18]. Historically, two to three hours was considered a safe upper limit for tourniquet use in upper extremity surgery [18,29,30]. In our study, we observed comparable mean inflation durations of 50.3 and 52.1 minutes in DRF patients with the tourniquet deflated before and after closure, respectively. A growing body of literature has formed around the use of WALANT in DRF surgery, with numerous studies finding equal or improved outcomes with regard to postoperative hospitalization and patient-reported pain immediately following surgery when compared to traditional tourniquet protocols [3,28], suggesting the WALANT approach may be a viable alternative in this patient population.

The results of this study must be considered in the context of its limitations. As a retrospective study at a single institution, our findings may not be representative of the broader population of patients undergoing CTR, TF release, and DRF procedures. Our findings may be influenced by inherent selection bias, as significant and unaccounted for baseline differences may exist between groups. Although we adjusted for patient and healthcare system factors in our multivariate analysis, other unmeasured confounding factors as well as non-random allocation of the groups may have influenced our results. Furthermore, specific technical details of the procedures performed were not analyzed, which may have influenced postoperative outcomes. Additionally, a potential bias was introduced by variable follow-up protocols among surgeons, where some surgeons do not require follow-up appointments if patients are recovering well following CTR and TF and others always schedule follow-up appointments. While this introduces a selection bias as only patients with documented follow-up were included in the study, this may have increased the probability of detecting differences between groups, thereby strengthening some of the study's findings rather than diminishing them. Moreover, the prescription of opioids in the PACU is influenced by the providers on staff and potentially multiple other patient-specific factors that were not controlled for. Finally, the study used a short follow-up period of 30 days postoperatively and had a relatively small sample size of 150 patients per procedure group, potentially leading to type II error. Therefore, the largely negative findings of the study should be interpreted with caution. Larger studies with longer follow-up periods are required to confirm this study's findings.

## Conclusions

This study's findings demonstrate that the timing of tourniquet deflation, either before closure or after closure, does not significantly impact the majority of early postoperative clinical outcomes or PROMs following CTR, TF, and DRF. While the overall outcomes were similar, procedure-specific differences in immediate recovery were observed. Notably, for CTR, deflating the tourniquet after closure was associated with increased opioid administration in the PACU. In contrast, for TF, the same after closure protocol was linked to significantly reduced pain scores, albeit with longer recovery times. For DRF, no significant differences were found between the two deflation protocols. These findings suggest that both deflation timings are safe and viable options for patients undergoing these procedures. The choice of when to release the tourniquet can be guided by surgeon preference and the specific context of the procedure. Further study is warranted to identify optimal tourniquet deflation timing protocols in common upper extremity surgeries.
